# Recent Developments in Macrophages Imbalance and Recurrent Spontaneous Abortion

**DOI:** 10.1155/jimr/8862057

**Published:** 2026-07-24

**Authors:** Yi Xiao, Fan-Yu Zeng, Jin-Song Liu, Feng Zhao, Jing-Li Sun

**Affiliations:** ^1^ Department of Obstetrics and Gynecology, General Hospital of Northern Theater Command, Shenyang 110000, Liaoning Province, China, syjqzyy.com; ^2^ Department of Research, General Hospital of Northern Theater Command, Shenyang 110000, Liaoning Province, China, syjqzyy.com; ^3^ Basic Medical College of China Medical University, Shenyang 110000, Liaoning Province, China

**Keywords:** factor, immune, M1/M2-type macrophages imbalance, recurrent spontaneous abortion

## Abstract

Recurrent spontaneous abortion (RSA), defined as two or more consecutive pregnancy losses, remains a significant clinical challenge in reproductive medicine. One of the primary reasons of RSA is abnormal immunological circumstances at the interaction between mother and fetus. Emerging evidence highlights the critical role of immune dysregulation, particularly involving macrophages, in the pathogenesis of RSA. Macrophages, key immune cells in the decidua, exhibit phenotypic plasticity and can polarize into pro‐inflammatory M1 or anti‐inflammatory M2 subtypes, thereby influencing trophoblast invasion, placental development, and immune tolerance. How the immune system’s macrophages balance impacts an embryo’s development is therefore essential to study. This review elaborates on the mechanisms by which alterations in transcription factors, signaling pathways, cytokine profiles, epigenetic status, and metabolic reprogramming contribute to pregnancy loss. Such aberrations predominantly drive the polarization shift toward the M1 macrophage phenotype, characterized by an elevated M1/M2 ratio. This imbalance triggers excessive secretion of pro‐inflammatory cytokines, impairs trophoblast invasion, and disrupts placental angiogenesis, thereby ultimately inducing adverse pregnancy outcomes and spontaneous abortion. By integrating findings from human studies and animal models, this review aims to provide insights into the immune mechanisms underlying RSA and highlights future research directions for improving clinical outcomes.

## 1. Introduction

Three or more embryonic damages, including biochemical pregnancy, before 28 weeks of pregnancy are typically defined as recurrent spontaneous abortion (RSA). The probability of recurrence has, however, grown dramatically in recent years following two consecutive spontaneous abortions (SAs), and the etiological component ratio of the second and third SAs is comparable. Expert consensus indicates that pregnancy loss, including biochemical pregnancy, with the same partner for two or more consecutive times prior to the 28th week of pregnancy is classified as RSA given the fact that the number of advanced‐age pregnant women grow annually [1, 2]. The reasons for RSA are numerous. Multiple studies on reproductive immunology conducted in the past few years have demonstrated a significant connection between the abnormalities of the milieu at the mother–fetus interaction and the development of RSA.

Throughout the whole gestation process, the different cells and cytokine groups that make up the immune milieu between mother and fetus are vital. Macrophage, the second largest kind of immune cells in maternal‐fetal interface, can influence the environment around maternal‐fetal interface. M1‐type macrophages are classically activated macrophages that can generate proinflammatory substances to intensify immune response. To keep immune response, M2‐type macrophages are alternatively activated macrophages, which generate various anti‐inflammatory substances. Throughout gestation, the equilibrium of M1/M2‐type macrophages is crucial for the immunological response. Because of the disruption of the maternal‐fetal contact microenvironment caused by this abnormal cell balance, immunological tolerance cannot be preserved, which results in RSA. It is discovered that macrophage imbalance is caused by modifications to transcription factors, associated signal pathways, cytokines, and enzyme‐related factors. This research presents the immunological mechanism of RSA induced by the imbalance of macrophages in the immune milieu of the maternal‐fetal interface, along with recommendations for future prevention and treatment.

## 2. Immune Landscape at the Maternal‐Fetal Interface

The maternal‐fetal interface exhibits a unique immune tolerance phenotype, balancing semiallogeneic fetal antigen acceptance and local immune defense to prevent rejection while resisting pathogens [3]. This specialized tissue microenvironment is structurally and functionally constituted by decidual stromal cells, trophoblast cells, and a distinctive local leukocyte repertoire, including uterine natural killer (NK) cells, macrophages, and regulatory T cells, which jointly shape the immunosuppressive and immune‐homeostatic microenvironment for successful pregnancy [4].

Within the intricate system of immune homeostasis, a multitude of cells perform specialized functions and maintain mutual checks and balances, collectively forming a comprehensive regulatory network that spans from innate to adaptive immunity and encompasses both immune activation and suppression.

The immune landscape is shaped by the specialized functions and reciprocal regulation of key cellular players. While both macrophages and dendritic cells (DCs) present antigen, their core missions differ: macrophages focus on phagocytic clearance and tissue homeostasis, whereas DCs excel at migration and T‐cell priming to initiate adaptive immunity [5–8]. NK cells complement this by providing innate surveillance against compromised host cells [9, 10].

The adaptive response is further polarized by the counteracting roles of Th17 and regulatory T (Treg) cells—the former driving protective but potentially pathogenic inflammation via IL‐17 and the latter maintaining tolerance via IL‐10 and TGF‐*β* [11–13].

Macrophage polarization into M1 or M2 phenotypes introduces a decisive environmental cue, profoundly influencing the Th17/Treg equilibrium. M1‐associated cytokines (e.g., IL‐6 and IL‐23) push T cell differentiation toward the Th17 lineage, while M2‐derived factors (e.g., IL‐10 and TGF‐*β*) support Treg development. This regulatory circuit is bidirectional: Th17 cells can amplify M1 responses, while Tregs can suppress macrophage activation or steer them toward an M2‐like, proresolving phenotype. Thus, the interplay between macrophages, Th17, and Treg cells constitutes a central self‐regulatory module that balances immune activation and suppression, with dysregulation underpinning various inflammatory diseases and cancer [14, 15].

## 3. Macrophages

Macrophages, as ubiquitous cellular components in the body, are distributed throughout all tissues and play a key role in maintaining homeostasis. They dynamically respond to a wide range of internal and external signals, exhibiting high plasticity that allows them to adapt their phenotypes according to changes in the environment [16, 17]. Decidual macrophages (dM*φ*s) are the predominant immune cells which are present at the interaction between mother and fetus, and they are the group of myometrial leukocytes. Their quantity is second only to NK cells, and they serve as crucial for angiogenesis, immunology control, and tissue regeneration [18]. dM*φ*s exhibit unique functions at each stage of pregnancy, which are facilitated by their phenotypic plasticity [19, 20]. As pregnancy progresses, the number of dM*φ*s increases, highlighting their essential role throughout gestation [21]. Two subtypes of macrophages can be found in tissues: classically activated macrophages (M1‐type) and alternatively activated macrophages (M2‐type). M1 macrophages can be identified by surface markers such as cluster of differentiation (CD) 80, CD86, and HLA‐DR. The costimulatory molecules CD80 and CD86 reinforce the functional identity of M1‐polarized macrophages as pivotal orchestrators of adaptive immune activation. Conversely, M2 macrophages express markers like CD163, CD206, and CD209. The mannose receptor (CD206) serves as both a defining marker and an effector mechanism of the IL‐4/STAT6‐mediated alternative (M2) activation pathway, directly contributing to its immunoregulatory and tissue‐remodeling functions [22–24]. In some circumstances, M1‐type macrophages may accelerate the detrimental inflammatory process. The four subgroups of M2‐type macrophages (M2a, M2b, M2c, and M2d) are all demonstrated to be immunosuppressive properties. M1‐type macrophages are pro‐inflammatory in nature that can destroy intracellular microbes and trigger Th1 response due to their enhanced antigen‐presenting capacity. M2‐type macrophages stimulate Th2‐ or antibody‐mediated immune responses, contribute to tissue remodeling, and have the capacity to suppress the immune system [8, 25–27]. The ratio of macrophage subtypes and the number of cytokines vary throughout every stage of pregnancy, whereas macrophages maintain a dynamic equilibrium during an ordinary pregnancy. Scavenger receptors confer phenotypic plasticity to macrophages, enabling context‐dependent responses: engagement by pathogen‐ or damage‐associated molecular patterns promotes proinflammatory signaling consistent with M1 polarization, whereas recognition of apoptotic cells facilitates efferocytosis and induces resolution‐promoting, M2‐like functional states. The overall polarization outcome is determined by the integration of signaling inputs across simultaneously engaged receptor networks [23, 24].

## 4. The Balance of M1/M2‐Type Macrophages in Pregnancy

The quantity and proportion of M1‐type and M2‐type macrophages at the mother‐fetus interface vary during every stage of a typical pregnancy. M1‐type tends to polarize dMφs prior to blastocyst implantation. After implantation, mixed M1/M2‐type macrophages make up the majority of the cell population, especially when the trophoblast begins to penetrate the myometrium. To safeguard both the embryo and placenta until delivery, dMφs change into the predominant M2‐type in the latter stages of placental development. During this period, the focus is on maintaining maternal‐fetal tolerance to ensure that the interaction between maternal and fetal tissues does not trigger immune rejection. As parturition approaches, the immunoregulatory role of dM*φ*s shifts, triggering a proinflammatory response that initiates spontaneous labor. Once a maladaptation in either the maternal or fetal immune system causes a shift in immune function, it can lead to placental developmental defects and a disproportionate inflammatory response toward fetal and placental tissues [28, 29]. At the maternal‐fetal interface, dMφs serve as key antigen‐presenting cells that sample and process fetal antigens, presenting them via MHC class II molecules to local T cells. In normal pregnancy, these macrophages predominantly exhibit an M2‐like phenotype, secreting anti‐inflammatory cytokines such as IL‐10 and TGF‐*β* to maintain immune tolerance, support tissue remodeling, and promote angiogenesis. However, in RSA, macrophage M1/M2 polarization is disrupted, with a shift toward a pro‐inflammatory M1‐dominant state characterized by elevated expression of CD80/CD86, enhanced antigen‐presenting activity, and excessive secretion of TNF‐*α* and IL‐1*β*, ultimately leading to breakdown of maternal‐fetal immune tolerance.

**Figure 1:**
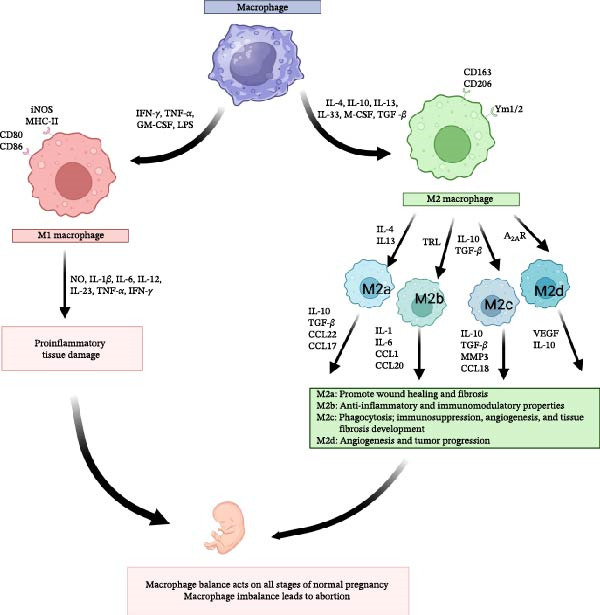
Macrophages and their related factors participate in all stages of pregnancy. M1 macrophages are induced by IFN‐*γ*, TNF‐*α*, GM‐CSF, and LPS, whereas M2 macrophages are actived by IL‐4, IL‐10, IL‐13, IL‐33, M‐CSF, and TGF‐*β*. M1 macrophages have cell markers in CD80, CD86, iNOS, and MHC‐II, whereas M2 have CD163, CD206, and Ym1/2. M1 is associated with Th1 response, including cytokine production of NO, IL‐1*β*, IL‐6, IL‐12, IL‐23, TNF‐*α*, and IFN‐*γ*. M2 leads to Th2 response, including cytokine production of IL‐10, TGF‐*β*, and VEGF. The quantity and proportion of M1‐type and M2‐type macrophages at the mother‐fetus interface vary during every stage of a typical pregnancy. M1/M2‐type macrophages imbalance at the mother and fetus interaction could lead to a rise in immunological rejection, which would hinder the establishment of immune tolerance and ultimately cause RSA. The figure was created by BioRender.

Relevant studies have shown that in the decidua tissue of RSA patients, the percentage of M1‐type macrophages is higher, macrophage autophagy is improved, and the percentage of M2‐type macrophages decreases [25, 30]. In their single‐cell analysis, dMφs were characterized into two subtypes—M1 gene‐enriched mac1 and M2 gene‐enriched mac2. A shift in their balance was observed in RSA, with an increase in mac1 and a significant decrease in mac2 cells [31, 32]. The way we define macrophage imbalance is by comparing the proportions of M1/M2‐type cells. It suggests that a mismatch in M1/M2‐type macrophages at the mother and fetus interaction could lead to a rise in immunological rejection, which would hinder the establishment of immune tolerance and ultimately cause RSA (Figure 1).

## 5. Mechanism of RSA Caused by an Imbalance in Macrophages

### 5.1. Abnormal Expression of Transcription Factors

#### 5.1.1. Nuclear Receptor Rev‐Erb*α* (NR1D1)

Nuclear receptor Rev‐erb*α* (NR1D1), also known as nuclear receptor subfamily 1, group d member 1, is a transcription regulator that controls macrophage gene expression in addition to the circadian rhythm [33]. According to the recent researches, patients who underwent abortion had greater amounts of M1‐type macrophages in decidua but lower levels of NR1D1 expression. When NR1D1 was knocked out, macrophages polarized M1‐type; however, via activating the PI3K/Akt signaling pathway, Sr 9009, an agonist of Rev‐erb *α*, caused M2‐type macrophages to become polarized [34, 35]. These offer an achievable objective for minimizing pregnancy loss and complications while additionally confirming Rev‐erb*α*’s regulatory effect on macrophage development and activity (Table 1).

**Table 1:** The immunological mechanism of RSA caused by the imbalance of macrophages.

Class	Title	Function	Reference	Relevance of RSA
Abnormal expression of transcription factors	NR1D1	Circadian rhythm, macrophage gene expression	CuCui et al. [33]	↓ NR1D1 → M1↑
	NF‐*κ*B	Master regulator of inflammation, immune response modulator, key effector pownstream of pathways	Brasier [36] Ohtsu et al. [37]	↓ NF‐*κ*B → M1↑
Dysregulated signaling pathways	PD‐1 and PD‐L1	Immune damage, immune tolerance and the balance of decidual macrophages	Zhang et al. [38]	↓ PD‐1 → M1↑
	Tim‐3 and Gal‐9	Release inhibitory signals to polarize macrophages	Zhu et al. [39] Li et al. [40]	↓ Tim‐3/Gal‐9 → M1↑
Cytokines imbalance	G‐CSF	Derive from M2‐type macrophages, trophoblast invasion and migration	Svensson et al. [41]	↓ G‐CSF ← M2↓
	VEGF	Angiogenesis and vascular permeability, a high predictive value for RSA in early pregnancy	Zhonghui and Qiuyu [42] Zhao et al. [43]	↓ VEGF → M2↓
	TGF‐*β*1	Cell invasion and migration, the development of cell homeostasis, trophoblast adhesion and invasion, maternal‐fetal rejection and embryo implantation	Yang et al. [44] Singh et al. [45]	↓ TGF‐*β*1 → M2↓
Aberrant epigenetic landscapes	miRNAs	Regulate immune cell function and inflammatory responses	Zhang [46]	↑ miRNAs → M1↑
	HDAC8	Embryonic development, macrophages	Das Gupta et al. [47] Samanta et al. [48]	↓ HDAC8 → M2↓
	Cathepsine E	Expressed in immune system cells, apoptosis, angiogenesis, immune system, M1‐type macrophage inflammation	Zaidi and Kalbacher [49] Shin et al. [50]	↓ Cathepsine E → M1↓
	STMN1	Cell proliferation, differentiation, activity	Zaidi and Kalbacher [49]	↓ STMN1 → M1↑
	MMPs/TIMPs	Matrix rebuilding, the degree of invasion	Cabral‐Pacheco et al. [51]	↑ MMPs/↓ TIMPs → M1↑
Metabolic reprogramming abnormality	IDO	Polarize process of macrophages, immune tolerance	Huang et al. [52]	↓ IDO → M2↓
	iNOS	Surface indicator of M1‐type macrophages, ovulation, implantation, trophoblast invasion and embryo growth	Boldeanu et al. [53] Maul et al. [54]	↑ iNOS → M1↑

#### 5.1.2. Nuclear Factor‐*κ*B (NF‐*κ*B)

NF‐*κ*B constitutes a family of structurally homologous inducible transcription factors, with its signaling cascade serving as a principal driver of inflammatory responses [36]. The receptor for advanced glycation end products (RAGE), a member of the immunoglobulin superfamily, perpetuates inflammatory states through positive feedback mechanisms [55]. Upon ligand binding, RAGE activates the NF‐*κ*B signaling pathway, whose subsequent induction stimulates the production of diverse proinflammatory cytokines, chemokines, and reactive oxygen species (ROS), thereby promoting macrophage polarization toward the M1 phenotype [37, 56]. Human embryonic trophoblast cells and maternal decidual stromal cells release the TNF superfamily, including the receptor activator of nuclear factor‐*κ*B (NF‐*κ*B) ligand (RANKL). In order to preserve immune tolerance at the interaction between mother and fetus, dMφs are further stimulated to polarize into the M2‐type by stimulating the Akt/STAT6 signal and increasing the transcription of IRF4 and Jmjd3 [57]. By boosting macrophage adherence and expression to the decidual atmosphere, RANKL activates the NF‐*κ*B signaling that contributes to the recruitment and retention of dMφs [58]. Fetal loss and dMφ dysfunction may result from abnormally low levels of RANKL and adhesion molecules [59]. M2‐type macrophage polarization can be generated by the normal expression of RANKL.

### 5.2. Dysregulated Signaling Pathways

#### 5.2.1. The Pathway of PD‐1 and PD‐L1

The programmed cell death protein 1 (PD‐1) and its primary ligand, programmed death‐ligand 1 (PD‐L1), constitute a critical immunoregulatory axis essential for maintaining peripheral tolerance and modulating immune responses. PD‐1, expressed on activated T cells, B cells, and NK cells, functions as a key immune checkpoint receptor [60]. Its interaction with PD‐L1, which is constitutively expressed on antigen‐presenting cells and can be induced on various nonhematopoietic cells. The pathway of PD‐1 and PD‐L1 delivers a potent coinhibitory signal [61]. This interaction triggers the recruitment of phosphatases, such as SHP‐2, to the cytoplasmic tail of PD‐1, leading to the dephosphorylation of proximal signaling molecules in the T‐cell receptor cascade. Consequently, it suppresses T‐cell proliferation, cytokine production, and cytotoxic activity, effectively inducing a state of T‐cell exhaustion or anergy [62–64]. Scholars have shown in recent years that the pathway of PD‐1 and PD‐L1 can simultaneously reprograme metabolism through the PI3K/AKT/mTOR and MEK/ERK pathways, and they are important for the growth and operation of pregnant macrophages [65]. The main regulator of macrophage polarization and function, which are essential for the health of gestation, is the PD‐1/PD‐L1 signaling pathway. For instance, the lack or reduction of PD‐1 signal might cause macrophages to become M1‐type, decrease phagocytosis, increase glycolysis, secrete more proinflammatory cytokines, disturb the macrophage balance, and accelerate the rate of embryo absorption [38, 66].

#### 5.2.2. The Pathway of Tim‐3 and Gal‐9

In addition to negatively regulating T cell response, T cell immunoglobulin mucin‐3 (Tim‐3) can induce mother‐fetus immunological tolerance and express on macrophages. The occurrence of cancers, persistent viral infections, autoimmune disorders, and pregnancy complications are associated with a disability in Tim‐3’s expression and function [39]. Tim‐3 releases inhibitory signals through the pathway of Tim‐3/Gal‐9 after binding to its ligand, galectin‐9 (Gal‐9). It promotes the polarization of M1‐type macrophages, leading to an imbalance of M1/M2‐type macrophages due to the fact that a significant number of macrophages and inflammatory granulocytes congregate at the mother‐fetus contact. Researchers found that dMφs and peripheral blood mononuclear cells (PBMCs) in the RSA group had abnormal expression of Tim‐3 and Gal‐9, as well as atypical M1/M2‐type macrophage numbers [40, 67–69]. These demonstrate that the occurrence of adverse pregnancy results is linked to the suppression of the Tim‐3/Gal‐9 pathway, which can change the macrophage balance and result in abnormal immunological tolerance at the maternal–fetal interaction.

### 5.3. Cytokines Imbalance

#### 5.3.1. Granulocyte Colony Stimulating Factor (G‐CSF)

G‐CSF is crucial at different stages of pregnancy. G‐CSF from M2‐type macrophages can stimulate the pathway of PI3K‐AKT‐ERK, which in response can facilitate trophoblast invasion and migration [41]. Additionally, trophoblast colony stimulating factor (CSF) receptor can bind to G‐CSF, which is advantageous for maintaining the typical course of pregnancy. In comparison to normal pregnancy, RSA patients’ early pregnancy chorionic tissue exhibited considerably decreased levels of G‐CSF expression [70, 71]. The decreased G‐CSF expression impacts the ability of trophoblasts to proliferate and migrate, which also lessens the trophoblasts’ ability to suppress macrophages [72–74]. These validate that the aberrant expression of G‐CSF disrupts the M1/M2‐type macrophage balance, ultimately leading to a negative pregnancy outcome. Scholars both domestically and internationally have suggested using G‐CSF immunotherapy in the management of RSA; however, additional research is required to provide further support because there is now insufficient information on different doses and durations.

#### 5.3.2. Vascular Endothelial Growth Factor (VEGF)

VEGF contributes to the development of the vascular system. The majority of cells on the maternal‐fetal interface are able to secrete VEGF, which has the ability to simultaneously accelerate angiogenesis and regulate vascular permeability. It also has a high predictive value for RSA in the initial stage of pregnancy [42, 43]. Additionally, VEGF can encourage M2‐type macrophages polarization and macrophages recruitment in decidua [75]. The experimental group observed a decrease in embryo implantation rate through the inhibition of endometrial macrophages, and the expression of VEGF significantly decreased [76, 77]. These suggest that the balance of macrophages is related to the normal process of pregnancy and that low levels of VEGF have an impact on angiogenesis, which in consequently hinder angiogenesis and reduce the polarization of M2‐type macrophages.

#### 5.3.3. Transforming Growth Factor *β*1 (TGF‐*β*1)

M2‐type macrophages have the capacity to release TGF‐*β*1, which can facilitate cell invasion and migration and promote the development of cell homeostasis [44]. TGF‐*β*1 can enhance trophoblast adhesion and invasion, lessen maternal‐fetal rejection, and boost embryo implantation [45]. TGF‐*β*1 has a steady function during pregnancy, and an important risk indicator for both unsuccessful embryo implantation and secondary RSA is a reduction in TGF‐*β*1 expression.

### 5.4. Aberrant Epigenetic Landscapes

#### 5.4.1. MicroRNAs (miRNAs)

miRNAs are small noncoding RNAs that regulate gene expression at the posttranscriptional level [46]. Recently, growing evidence has implicated miRNAs in the polarization of M1 macrophages. For instance, miR‐103 has been identified as a potential diagnostic biomarker for RSA. STAT1/IRF1 signaling‐mediated M1 polarization can be targeted to protect against embryo loss in contexts of miR‐103 overexpression [78]. Additionally, miR‐194‐5p negatively regulates TRAF6 expression by directly targeting it, leading to suppression of both NF‐*κ*B and Wnt signaling pathways. This ultimately inhibits the proliferation, migration, and release of pro‐inflammatory cytokines such as IL‐6 and TNF‐*α* in M1 macrophages while also promoting their apoptosis [79]. Furthermore, extracellular vesicles derived from M1 macrophages deliver miR‐146a‐5p and miR‐146b‐5p, which suppress trophoblast migration and invasion in RSA by targeting TRAF6, thereby exacerbating pregnancy loss [80]. Similarly, exosomes from macrophages in RSA cases transfer miR‐153‐3p to trophoblasts, where it targets the IDO gene and inhibits cell proliferation and migration via the STAT3 pathway, contributing to the pathogenesis of RSA [81]. These results suggest that miRNAs expression profiles may serve as a basis for predicting RSA and inform the development of future treatment strategies.

#### 5.4.2. Histone Deacetylase 8 (HDAC8)

Histone‐modifying enzymes are known as histone deacetylases (HDACs). HDAC8 is thought to be intimately associated with the early stages of mammalian embryonic development, which may preserve a healthy pregnancy by activating macrophages, upregulating the expression of the M2 marker gene, and preventing macrophages death at the interaction between mother and fetus [47, 48]. The expression of HDAC8 and M2‐type macrophages count is both sharply decreased in RSA group’s [82, 83]. It has been established that HDAC8 expression can facilitate M2‐type macrophage polarization. This implies that inhibiting HDAC8 expression may cause M1/M2 macrophages to lose balance, which could lead to an unfavorable pregnancy outcome.

#### 5.4.3. Cathepsine E

As a protease that breaks down aspartic acid in the body, cathepsine E is mostly expressed in immune system cells [49]. By triggering apoptosis, suppressing angiogenesis, boosting the immune system, and contributing to M1‐type macrophage inflammation, it inhibits tumor growth and metastasis [50]. According to a study, cathepsin E was expressed in human decidua, including dMφs, and the RSA group’s cathepsin E secretion and activation were significantly decreased. This indicated a link between abortion and a reduction in cathepsin E activity [25, 84]. The results showed that a reduced amount of cathepsin E most likely caused the imbalance of M1/M2‐type macrophages, disturbance of normal mother‐fetus immunological tolerance, and proper trophoblast invasion, all of which led to abortion.

#### 5.4.4. Stathmin‐1 (STMN1)

As an intermediary for various intracellular signaling pathways, cytoplasmic phosphoprotein‐1 (STMN1) is regarded as a tiny regulatory protein that regulates cell proliferation, differentiation, and activity [49]. M1‐type macrophages may effectively block tumor necrosis factor *α* (TNF‐*α*)‐induced STMN1 expression in trophoblast cells. According to a study, at the first 3 months of pregnancy, TNF‐*α* expression dramatically rose, while STMN1 expression was downregulated in the cytotrophoblast and villi of RSA patients. STMN1 knockout successfully prevented decidualization and dramatically reduced trophoblast cell invasion and proliferation [8, 85]. This reveals that in early normal pregnancy, STMN1 production is upregulated at the embryo implantation site, which could facilitate endometrial decidualization while stimulate trophoblast migration and invasion [86]. Trophoblast infiltration and proliferation are impaired, and the interface’s environment between mother and fetus is altered, and STMN1 expression is decreased due to an imbalance in macrophage balance.

#### 5.4.5. Balance of Metalloproteinases (MMPs)/Tissue Inhibitors of MMPs (TIMPs)

A category of zinc‐dependent endopeptidases known as matrix metalloproteinases (MMPs) preferentially breaks down different extracellular matrix (ECM) constituents. It has been discovered that macrophages encourage intravascular trophoblast invasion and break down ECM by secreting MMPs [87]. The activity of MMPs can be controlled by TIMPs. In embryonic trophoblasts and decidua, the balance of MMPs and TIMPs expression can preserve matrix rebuilding and regulate the degree of invasion [51]. Through examinations, the researchers verified that the activation and production of MMPs could be induced by macrophages and other inflammatory cytokines. There had limited expression of TIMPs in RSA, nevertheless had significant expression of MMP‐2 and MMP‐9 [88, 89]. The outcome of pregnancy is bound to be affected by the imbalance between MMPs and TIMPs, and such an imbalance might result in aberrant tissue remodeling and ultimately pregnancy failure.

### 5.5. Metabolic Reprogramming Abnormality

#### 5.5.1. Indoleamine2,3‐Dioxygenase (IDO)

Tryptophan metabolism is triggered by the rate‐limiting enzyme, IDO. Studies had verified that in order to prevent immunological rejection, IDO expression at the mother–fetus interaction is needed [90]. The microenvironment around macrophages could be successfully improved by IDO expression, while the levels of IDO would influence the polarization process of macrophages [52]. The production of IDO in dMφs in the RSA group had a lower positive reaction rate than in the normal pregnancy group, indicating that abnormally low levels of IDO promote macrophages imbalance and raise the potential of abortion [91, 92]. This demonstrates that the positive IDO in dMφs with an M2‐dominant phenotype might promote trophoblast cell proliferation, prevent apoptosis in the early stages of pregnancy, preserve immunological tolerance at the maternal‐fetal interface, and diminish the risk of unfavorable pregnancy outcomes.

#### 5.5.2. Inducible Nitric Oxide Synthase (iNOS)

**Figure 2:**
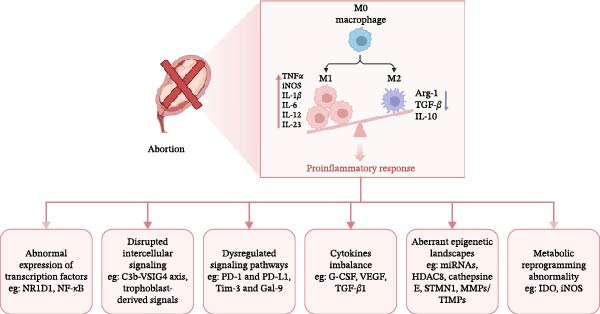
Mechanisms of macrophage polarization dyregulation in RSA. The figure was created by BioRender.

A particular surface indicator of M1‐type macrophages that can release more NO when triggered is iNOS. According to pertinent research, two different types of macrophages were present in abnormally substantial quantities, and the RSA group expressed more iNOS than normal [53, 65, 93]. Ovulation, implantation, trophoblast invasion, and embryo growth are all connected with normal expression of iNOS throughout an ordinary pregnancy. Excessive iNOS expression and NO release will pathologically trigger cytotoxicity, which will hinder oviduct activity, generate uterine smooth muscle contraction, and ultimately lead to abortion [54]. This clearly demonstrates how aberrant macrophages polarization is linked to aberrant iNOS expression, which impacts the immunological environment at the mother‐fetus contact and decreases immune tolerance (Figure 2).

## 6. Therapeutic Modulation

Currently, there are various immunotherapies available for RSA, which primarily involve allolmmune and autoimmune approaches. Allolmmune therapies mainly include intravenous immunoglobulin (IVIG), G‐CSF, and PBMC therapy. IVIG can reduce the activity of NK cells, enhance the function of regulatory T (Treg) cells, block anti‐HLA antibodies, and inhibit complement activation, thereby significantly improving the live birth rate in patients with RSA [94, 95]. G‐CSF promotes the synthesis of IL‐10 by Treg cells, facilitates transplantation tolerance, and consequently enhances endometrial remodeling and receptivity [96, 97]. PBMC therapy significantly modulates the maternal immune system by ameliorating the Treg/Th17 paradigm and regulating the expression of cytokines, transcription factors, and miRNAs associated with Treg and Th17 cells [98]. These immunotherapies mainly exert immune tolerance effects by regulating the proportion of immune cells at the maternal‐fetal interface. Autoimmune therapies consist of TNF‐*α* inhibitors, hydroxychloroquine, aspirin, and heparin [99–101]. TNF‐*α* inhibitors suppress immune responses, downregulate the activity of transcription factors, proteases, and protein kinases, and reduce the release of pro‐inflammatory cytokines, chemokines, and adhesion molecules [102]. These inhibitors have been utilized in the treatment of RSA to mitigate immune rejection rates [103]. Given the diversity of immunotherapeutic strategies for RSA, personalized treatment approaches are essential.

## 7. Summary and Prospect

A positive atmosphere at the interface between the female reproductive system and fetus is essential for a successful pregnancy. The development and preservation of pregnancy depend heavily on macrophages, the second most common type of immune cell. Throughout pregnancy, macrophages have been shown to exhibit varying degrees of M1/M2 polarization plasticity, and their dynamic balance acts as a stabilizing factor. The expression of factors related to macrophages is closely linked to the development as well as persistence of immunological tolerance at the mother‐fetus interface. It is crucial to investigate the immunological mechanism of macrophages and associated factors impacting pregnancy since, in addition to RSA, an imbalance of M1/M2‐type macrophages can lead to preeclampsia, fetal growth restriction, premature delivery, and other pregnancy problems. Though the mechanism of RSA caused by macrophages imbalance is currently ambiguous and requires more investigation, a wide array of therapeutic modulation is available, allowing for individualized treatment based on the relevant immunological mechanisms. Future research must focus on unraveling the dynamic interplay between established transcriptional, metabolic, and epigenetic regulators in shaping cellular polarization states. Deciphering these interconnected molecular networks will further highlight the inherent complexity of combinatorial therapeutic approaches while underscoring their potential to deliver mechanistic insights that inform targeted interventions and improved clinical management of RSA [71].

**Nomenclature**
RSA: Recurrent spontaneous abortion
SAs: Spontaneous abortions
M1‐type macrophages: Classically activated macrophages
M2‐type macrophages: Alternatively activated macrophages
NR1D1: Nuclear receptor Rev‐erb*α*
PPAR *γ*: Proxisome proliferator‐activated receptor *γ*
RANKL: Ligands of NF‐*κ*B receptor activator
PD‐1: Pogrammed cell death molecule‐1
PD‐L1: Pogrammed cell death molecule‐1 ligand
Tim‐3: T cell immunoglobulin mucin‐3
Gal‐9: Glectin‐9
G‐CSF: Granulocyte colony stimulating factor
VEGF: Vascular endothelial growth factor
TGF‐*β*1: Transforming growth factor‐*β*1
HDACs: Histone deacetylase 8
TNF‐*α*: Tmor necrosis factor *α*
STMN‐1: Stathmin‐1
MMPs: Metalloproteinases
TIMPs: Tissue inhibitors of MMPs
IDO: Indoleamine2,3‐dioxygenase
iNOS: Inducible nitric oxide synthase.

## Author Contributions

Yi Xiao and Fan‐Yu Zeng authored the manuscript’s initial draft. Jin‐Song Liu and Feng Zhao contributed to review and editing. Jing‐Li Sun conceived, evaluated, and revised this paper.

## Funding

This study was supported by the Natural Science Foundation of Liaoning Province, China (Grant 2024‐MSLH‐525).

## Disclosure

The final manuscript has been read and approved by every author. After using DeepSeek tool, the authors reviewed and edited the content as needed and take full responsibility for the content of the publication.

## Conflicts of Interest

The authors declare no conflicts of interest.

## Data Availability

Data sharing is not applicable to this article as no datasets were generated or analyzed during the current study.
